# Climate anxiety, environmental attitude, and job engagement among nursing university colleagues: a multicenter descriptive study

**DOI:** 10.1186/s12912-024-01788-1

**Published:** 2024-02-20

**Authors:** Mohamed Hussein Ramadan Atta, Mohamed A. Zoromba, Heba E. El-Gazar, Ahmed Loutfy, Mahmoud Ahmed Elsheikh, Omnya Sobhy Mohamad El-ayari, Ibrahim Sehsah, Nadia Waheed Elzohairy

**Affiliations:** 1 Psychiatric and Mental Health Nursing Department, Faculty of Nursing, Alexandria University, Admeon Freemon St, Semoha, Alexandria City Egypt; 2 College of Nursing, Prince Sattam Bin Abdulaziz University, Kharj City, Saudi Arabia; 3 Psychiatric and Mental Health Nursing, Mansoura University, Mansoura City, Egypt; 4 Administration Department, Faculty of Nursing, Port Said University, Port Said City, Egypt; 5 Nursing Department, College of Health Sciences, University of Fujairah, Fujairah, UAE; 6 Pediatric Department, Faculty of Nursing, Beni-Suef University, Beni Suef City, Egypt; 7 Community Health Nursing Department, Faculty of Nursing, Cairo University, Cairo City, Egypt; 8 Psychiatric Nursing and Mental Health, Faculty of Nursing, Kafr Elsheikh University, Kafr Elsheikh City, Egypt; 9 Faculty of Nursing, MTI University, Cairo City, Egypt; 10 Psychiatric Nursing and Mental Health, Damanhur University, Damanhur City, Egypt

**Keywords:** Environmental attitude, Climate anxiety, Job engagement, Nursing university colleagues

## Abstract

**Background:**

Climate change, a pervasive global phenomenon, exerts discernible impacts on the physical, social, and psychological dimensions of well-being. The apprehension surrounding this complex environmental issue has reached a critical juncture, with over 76,000 individuals across more than thirty nations expressing profound levels of concern, characterizing their anxiety as either "very" or "extremely" pronounced. This surge in awareness regarding the potential consequences of climate change has given rise to an emergent and escalating challenge known as climate anxiety. This distinctive form of anxiety manifests through profound feelings of fear, helplessness, and despair elicited by the impending repercussions of climate change. Notably, the intersection of climate anxiety with occupational domains, particularly within the context of Nursing University Colleagues, suggests a nuanced relationship with job engagement, wherein the psychological responses to climate change may influence professional commitment and involvement.

**Aim of the study:**

To examine the correlation among Climate Anxiety, Environmental Attitude, and Job Engagement among Nursing University Colleagues comprising eight distinct nursing faculties.

**Design:**

A multicenter descriptive, cross-sectional research design study followed.

**Subject:**

Three hundred fifty-nine participants from the Centre, Delta, West, Suez Canal, and Upper regions of Egypt using a stratified random cluster sampling technique.

**Measurements:**

Social and health related to climate data structured questionnaire, climate anxiety scale, environmental attitude inventory, and job engagement scale.

**Results:**

The influence of demographics on climate anxiety, environmental attitude, and job involvement was not observed. Nevertheless, geographical variations emerged as a noteworthy factor. A statistically significant inverse correlation was identified between climate anxiety, job engagement dimensions, and the overall score of environmental attitudes.

**Conclusion:**

Climate anxiety was strongly associated with environmental attitudes and job engagement among nursing university colleagues. Higher climate anxiety is associated with a lower attitude towards the environment and decreased job engagement. Additionally, a higher attitude towards the environment is associated with decreased overall engagement in participants’ jobs.

**Implications:**

The study's patterns make it clear how important it is to provide targeted psycho-educational interventions to help reduce climate anxiety among the group of nursing university colleagues. The imperative lies not only in alleviating the immediate psychological distress associated with heightened climate anxiety but also in fostering adaptive coping mechanisms. By doing so, these interventions serve as instrumental tools in nurturing resilience, thereby fortifying the mental well-being of nursing professionals amidst the evolving landscape of climate-related concerns.

**Supplementary Information:**

The online version contains supplementary material available at 10.1186/s12912-024-01788-1.

## Introduction

Climate change, as a ubiquitous global phenomenon, manifests discernible repercussions across the triad of physical, social, and psychological dimensions of human well-being. The multifaceted and complex nature of this phenomenon renders it an intricate subject of scholarly investigation, inviting comprehensive exploration into its far-reaching effects on the intricate fabric of individual and collective human existence. More than 76,000 individuals from over thirty countries expressed significant anxiety related to climate change [1]. An increasing prevalence of climate anxiety is observed, as over half of Americans have moderate to high worries about the effects of climate change on their lives [2]. Similarly, climate change was the primary concern for young people up to 24 years old, according to a global survey by the United Nations Development Programme Worldwide [3].

Climate change refers to long-term alterations in atmospheric patterns, such as temperature, precipitation, and wind, that are primarily influenced by human activities such as deforestation and the use of fossil fuels [4]. While the physical consequences of climate change are well-established, there is growing recognition of its psychological impacts. Environmental changes can impact psychological well-being, leading to stress and anxiety [5].

The dynamic interplay with the formidable force of climate change gives rise to a nuanced realm of affective experiences, wherein climate emotions, notably anxiety, come to the fore. Climate anxiety is a distinct form of anxiety that is characterized by individuals experiencing intense feelings of fear, helplessness, and desperation in response to the changes witnessed in our climate system [6]. Importantly, it is essential to recognize that climate anxiety can have a significant influence on both the mental health and physical well-being of individuals experiencing it. In the previous literature, a positive correlation was discovered between traumatic incidents resulting from climate fluctuations and the heightened susceptibility to anxiety and emotional-based disorders, specifically the danger of developing traumatic stress disorder [7].

These traumatic experiences were found to contribute to annoyances in sleep patterns, increased morbidity, and mortality associated with mental illness, in addition to a rise in psychiatric emergencies [8]. Moreover, antecedent scholarly works have discerned a discernible positive correlation between anxiety attributed to climate change and environmental attitudes. The extant literature, steeped in academic rigor, has systematically illuminated the interconnection between heightened climate-induced anxiety and the formation of environmentally attitudinal orientations [9].

The concept of environmental attitude encompasses an individual's thoughts, emotions, and actions towards the natural environment. This includes their beliefs, values, feelings, and willingness to work actively for its preservation. It is widely recognized that attitudes toward climate change can have a significant impact on the development of effective solutions [9]. Previous research showed that people with a positive environmental attitude are more likely to recycle, reduce energy use, and use alternative transportation [10, 11]. In addition, there is a strong correlation between favorable environmental attitudes and the implementation of climate change mitigation strategies, as well as their impact on job performance and occupational functioning [12].

Job engagement refers to an individual's emotional and cognitive involvement in their job, characterized by high enthusiasm, dedication, and absorption in job tasks [13]. It reflects an employee's willingness to exert effort, often resulting in higher productivity, organizational commitment, and job satisfaction. The impacts of climate change on job engagement can be significant, as severe climate events such as floods, droughts, and storms can disrupt job routines, damage infrastructure, and lead to financial losses, which can undermine job engagement [14]. Based on the Job Demands-Resources Model, job resources, including psychological well-being, contribute to increased job engagement [15].

As universities play a critical role in anxiety related to climate change research, education, and policy development, ensuring the engagement, motivation, and commitment of faculty colleagues is crucial in promoting sustainability and addressing the multifaceted difficulties presented by climate change [16]. Considering that university professors frequently engage in both research and teaching related to climate change, they are likely to encounter climate anxiety—characterized by emotional distress arising from an awareness of climate change. Nevertheless, there is a notable gap in the extensive examination of the impact of climate anxiety on academics' environmental attitudes and their levels of job engagement [17]. This study aims to investigate how climate anxiety influences environmental attitudes and job engagement among university academics.

The World Bank reports that Egypt, being particularly vulnerable to climate change due to its geographical location and socio-economic structure, faces intensified implications [18]. This study’s objective is to investigate the complex interplay between climate anxiety, environmental attitudes, and job engagement among nursing university colleagues in Egypt using a descriptive multi-center approach.

A limited body of scholarly inquiry has delved into the intricate nexus between climate anxiety and environmental attitudes, with a notable absence of investigations specifically within the nursing sector in the Egyptian context. The lack of extensive academic research on this subject highlights the significance of conducting comprehensive and nuanced studies to understand the perspectives of nurses in Egypt regarding climate change and its impact on their attitudes toward the environment. The significance of this study lies in its unique integration of climate anxiety, environmental attitudes, and job engagement among academic nursing colleagues. While previous research has examined these variables independently or in different combinations, to the best of our knowledge, none have investigated them collectively within the healthcare sector, particularly among nursing staff in Egypt. Thus, this study addresses an important gap in the literature. Understanding how climate anxiety and environmental attitudes influence job engagement among academic nursing colleagues, who play a leading role in education related to patient care, could have significant implications for patient outcomes and healthcare delivery.

The impact of climate anxiety on job engagement is a relatively understudied area. Although studies have demonstrated that climate anxiety can influence various aspects of mental well-being, few have explored its potential impact on job engagement in the healthcare sector. This is a critical oversight, as climate anxiety could potentially affect the ability of healthcare professionals to remain engaged and effective in their jobs. Therefore, investigating this relationship within the context of Egypt's academic nursing colleague sector is both innovative and timely, providing valuable insights into how climate anxiety may influence job-related attitudes and behaviors in this important field.

Egypt is a country with a diverse history, geographical topography, and a rapidly increasing population that faces formidable challenges concerning climate change and sustainability. It is imperative to examine how these challenges impact the nursing profession in particular. As healthcare professionals, nurses play a critical role in addressing public health issues; thus, understanding how climate-related factors and environmental attitudes intersect with job engagement among nursing practitioners in Egypt is of utmost importance. The research aims to contribute to the scholarly knowledge base on this topic and inform targeted interventions and policies to enhance the resilience and well-being of nursing professionals in Egypt. Additionally, the study's outcomes can influence nursing education by emphasising the necessity of including climate change and environmental sustainability topics in curricula to equip nursing practitioners to address these challenges effectively.

Lastly, by adopting a multi-center descriptive approach, this research contributes to a deeper understanding of these phenomena. The effects of climate anxiety, environmental attitudes, and job engagement may vary significantly across cultural contexts due to differences in environmental experiences, cultural norms, and job attitudes. By examining these variables within the specific cultural context of Egypt, this study adds to the limited body of literature that explores these issues outside Western contexts. Consequently, this could inform culturally specific interventions that may be more effective in addressing climate anxiety and fostering job engagement among nursing staff in Egypt and potentially similar settings.

### Objectives of this research

- Identify the levels of anxiety related to climate change, environmental attitude, and job engagement among nursing university colleagues.
- Compare the means of anxiety related to climate change, environmental attitude, and job engagement among nursing university colleagues among the eight nursing faculties.
- Assess the role of anxiety related to climate change in the relationship between environmental attitude and job engagement among nursing university colleagues.

### Research questions

- What are the levels of anxiety related to climate change, environmental attitude, and job engagement among nursing university colleagues?
- Is there a significant difference in the mean levels of anxiety related to climate change, environmental attitude, and job engagement among nursing university colleagues across the eight nursing faculties?
- How does climate anxiety affect the relationship between environmental attitude and job engagement among nursing university colleagues?

## Materials and procedure

### Setting and Design

A multicenter descriptive, cross-sectional research design study followed the “Improving the reporting of observational studies in epidemiology” (STROBE) checklist (Supplementary 1). The study was conducted at eight nursing Egyptian faculties that represent diverse Egyptian cultures, with Cairo with MTI University representing the Centre for Egypt, Mansoura University, Damanhur University, and Kafr El-Sheikh University reflecting the Delta and lower demographic regions, Alexandria University representing the western region, Port-Said University affiliated with the eastern and Suez Canal regions, and Beni-Suef University affiliated with the upper Egypt region.

### Population and sample size

The current study targeted the population of all academic nursing staff enrolled in the eight selected faculties. Data was collected using a stratified random cluster sampling method. To determine the required sample size for the study, the researchers used Open Epi, Version 3, which is an open-source calculator. The total population size of nursing staff in the studied universities was 1504. The parameters used in the calculation were as follows: a hypothesized percentage frequency of the outcome factor in the population (p) of 50% with a ± 5% margin of error, a confidence level of 95%, a design effect (DEFF) of 1 (applicable for cluster surveys), and the formula for sample size calculation: Sample size (n) = [DEFF * N * p * (1-p)] / [(d^2 / Z^2) * (N-1) + p * (1-p)]. Based on these parameters, the minimum required sample size was determined to be 307. However, an additional 30% of colleagues were needed to account for potential non-response, the online data collection method, and the anticipated dropout rate [19]. Therefore, the final sample size required for the study was 399. The final complete responses were 359, with a 90% response rate.

### Sampling method

**Fig. 1:**
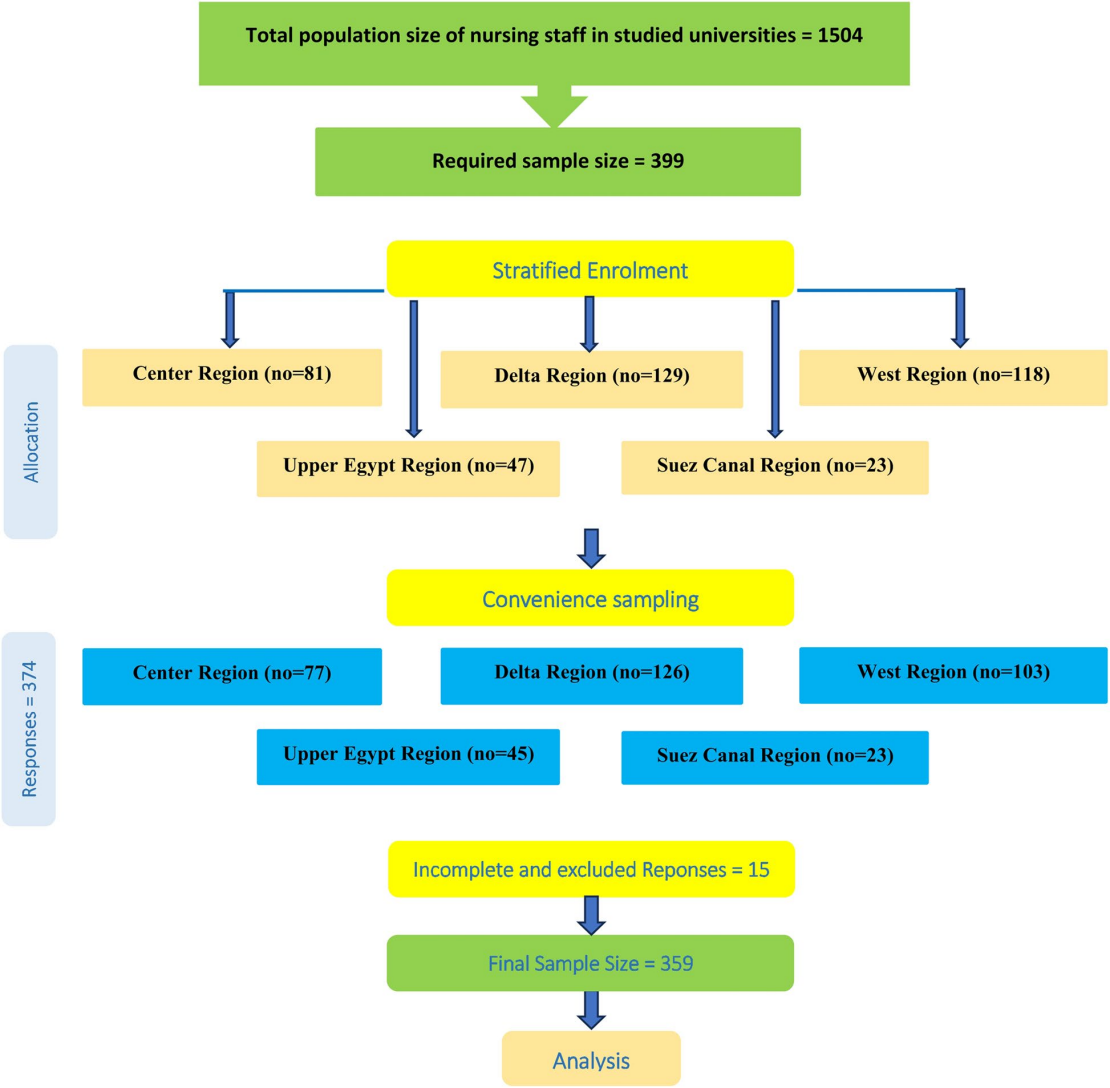
Sample graph

A two-phase sampling strategy was employed to enroll staff. Phase 1, stratified sampling was used to calculate the number of participating nursing staff per faculty. The targeted sample size from the faculties of Cairo, MTI, Mansoura, Damanhur, Kafr El-Sheikh, Alexandria, Port Said, and Beni-Suef was 62, 21, 67, 41, 21, 119, 22, and 47, respectively. The stratification approach was chosen to ensure that each faculty was adequately represented in the sample, accounting for variations in staff size and demographic characteristics across different universities and aiming to reduce sampling bias. In Phase 2, convenience sampling was used to recruit staff in each faculty; convenience enrolment was approached to facilitate practicality and feasibility in data collection and to mitigate the potential biases associated with this approach; and strict implementation of inclusion criteria was as follows: faculty member at least 6 months ago enrolment to ensure that participants have sufficient experience and exposure in their respective faculties to provide informed responses relevant to our study's objectives, faculty members actively involved in teaching and research activities, as they are directly engaged with the academic and environmental aspects of the universities, willingness to participate in the study and provide informed consent, ensuring voluntary participation and ethical compliance. Exclusion criteria included faculty members on extended leave (e.g., sabbatical, maternity, or medical leave) during the data collection period, as their absence might affect their perceptions and experiences related to the study objectives. Figure 1 shows a flow diagram describing the sampling method (Fig. 1 Sample Graph).

### Measurements of interest

After the collection of Social and Health-related Climate Change data through a structured questionnaire, three tools were applied. Information related to social profiles contains age, position, and department, as well as health-related profiles such as the presence of physical disease affected by climate change, the disease type, and the most probable cause of climate change (Supplementary 2).

- I. The Climate Anxiety Scale, created by Clayton and Karazsi (2020) [6], is a tool specifically designed to evaluate the psychological distress associated with climate change. It consists of 13 items that individuals rate on a scale ranging from 1 (never) to 5 (almost always). The total score is calculated by summing the scores of all items, with higher scores indicating greater climate anxiety. The scale encompasses two factors that capture different dimensions of climate anxiety: cognitive-emotional impairment and functional impairment. The cognitive-emotional impairment factor assesses the degree to which individuals experience psychological and emotional distress about climate change. In contrast, the functional impairment factor focuses on the effect of climate anxiety on individuals' everyday functioning and behavior. Through the current study, the scale was translated into Arabic and tested for reliability using a test–retest approach, yielding a Pearson coefficient of 0.74, indicating good reliability.

- II. Milfont and Duckitt (2010) [20] developed the environmental attitude inventory (EAI) as a psychometric tool to assess pro-environmental attitudes. It consists of 24 items divided into two subscales: Preservation and Utilization of the environment. The scores of each subscale are summed separately, with higher scores indicating stronger pro-environmental attitudes. Participants rate their agreement with each item on a 7-point Likert scale, ranging from 1 “Strongly disagree” to 7 “Strongly agree”.

The EAI has been demonstrated to be a valid and reliable instrument for assessing environmental attitudes. It has a high internal consistency, with a Cronbach's alpha coefficient of 0.83, indicating good reliability. Additionally, its structural validity has been confirmed, suggesting that it effectively captures the underlying dimensions of environmental attitudes [21]. The translated Arabic version in the current research was found to be reliable, with a test-retest correlation coefficient (Pearson's r) of 0.8, indicating good stability over time.

- III. Job Engagement Scale (JES) was established by Rich, LePine, and Crawford in 2010 [22]. It consists of 18 items that assess three dimensions of job engagement: physical, emotional, and cognitive engagement. A higher score on the scale indicates a higher level of job engagement. Participants rate each item on a scale extending from 1 (never) to 5 (almost always). In a study conducted by Huole et al. in 2021 [23], they applied a factor-ESEM (Exploratory Structural Equation Modeling) solution to the JES. The results showed that the factor loadings and composite reliability for all the factors were acceptable. The factors and their respective findings are as follows: Global engagement: The factor loadings ranged from λ = 0.563 to 0.814, with an average factor loading (Mλ) of 0.657. The composite reliability (ω) was 0. 970. Also, the reliability of the Arabic version of the JES was assessed by a Pearson correlation coefficient.

The study involved five experts in psychiatric nursing, and the reliability coefficient was found to be 0.79

Face validity was assessed through a panel of experts, including experienced nurses and academic professionals in environmental psychology and nursing. They reviewed the scales for appropriateness, relevance, and comprehensibility in the context of nursing university colleagues. Their feedback was used to confirm that the items were suitable and easily understandable for this specific population. Content validity was ensured by engaging a panel of experts in nursing and environmental studies. They evaluated the scales to ensure that the items comprehensively covered the aspects of climate anxiety, environmental attitudes, and job engagement relevant to the nursing colleagues. 

### Ethical consideration

Approval by the Research Ethics Committee (REC) was utilized, College of Nursing, Damanhur University, approval (IRB: 83-a). The study's objective was clearly communicated to the participants involved, ensuring that all data collected would be utilized solely for research reasons. Also, each participant was notified of their right to decline participation or withdraw from the study before finishing the study materials without facing negative repercussions. The study sought informed written consent from nursing colleagues who agreed to participate. The consent process was conducted online, with each participant confirming their consent electronically before proceeding to the survey. To maintain confidentiality and data security, the online platform anonymized responses, ensuring that data was used exclusively for research purposes. Access to this data was restricted to the research team only.

### Data collection phase

The current study utilized an online method for data collection, motivated by the ease of access for participants across multiple university campuses, logistical efficiency, and environmental benefits of reducing paper use. Research instruments were transformed into an online format using a secure, user-friendly survey platform (Google Forms). A pilot study involving 25 individuals from a Nursing University was conducted to test the survey's functionality and clarity. Following formal approval from relevant authorities, email invitations containing the survey link were sent to potential participants from the eight selected faculties. Data collection occurred from July 2023 to August 2023, with participants given a specific timeframe to complete the survey, complemented by periodic reminders. After the collection period, the responses were downloaded and analyzed statistically, as detailed in our manuscript.

### Statistical analysis

The acquired data was aggregated and presented in tabular form. Variables with a continuous distribution that deviated from normality were represented using the median and Interquartile Range (IQR). On the other hand, categorical variables were presented in the form of numerical counts and corresponding percentages. The researchers employed the Spearman two-tailed correlation coefficient to evaluate the association between Climate Anxiety, Environmental Attitude, and Job Engagement. The interpretation of the correlation coefficient involved categorizing it as representing either a low, moderate, or strong association. The hierarchical multiple regression method was used to assess the impact of climate anxiety on job engagement and then to add environmental attitude, thus illustrating each variable's unique contribution and their combined effect. This approach also facilitated controlling for confounding variables, ensuring that the unique impact of each predictor was accurately isolated. Additionally, it quantitatively evaluates the incremental variance in job engagement explained by environmental attitude after accounting for climate anxiety. The determination of statistical significance on *p*-values ≤ 0.05 The statistical analyses were conducted utilizing IBM Corp.'s SPSS application, version 27.

## Results

Table 1 revealed a diverse age representation, with the largest proportion of participants (49.3%) being between 30 and less than 40 years. The data suggested that different age groups did not show significant differences in terms of climate anxiety, EAI, and JES scores. Similarly, when examining the data based on sex, it was observed that a majority (77.4%) of the participants were female, yet no significant gender-based differences were found in the scores of the three measured variables. The marital status of participants was also considered, showing a predominance of married individuals (69.9%). However, marital status did not significantly affect the scores of climate anxiety, EAI, and JES. In terms of residence, the majority resided in urban areas (67.7%), but again, no significant differences were noted between urban and rural residents concerning the study variables.

**Table 1:** Participants’ demographics and differences in Climate anxiety, EAI, and JES

Demographics	Category	No	%	Climate Anxiety		EAI		JES	
				Mean (SD)	Sig	Mean (SD)	Sig	Mean (SD)	Sig
Age (years)	Less than 30 years	100	27.9%	29.5 (5.7)	.443	111.6 (11.5)	.134	66.7 (14.5)	.117
	From 30 to less than 40 years	177	49.3%	29.3 (5)		112.3 (14.1)		65.5 (15.3)	
	From 40 to less than 50 years	46	12.8%	30 (6.2)		113 (13.8)		65.5 (15.6)	
	50 years and more	36	10.0%	30.3 (4)		112.3 (14.2)		59.6 (15.9)	
Sex	Male	81	22.6%	29.2 (4.4)	.718	110.9 (14.4)	.980	66 (18.3)	.534
	Female	278	77.4%	29.6 (5.5)		112.6 (13)		65 914.3)	
Marital status	Single	78	21.7%	30.2 (5.7)	.310	112.1(14.1)	.942	64.1 (15.9)	.453
	Married	251	69.9%	29.3 (5.2)		112.3 (13.1)		65.3 (15.5)	
	Divorced Widowed	30	8.4%	30.3 (4.2)		111.5 (13.8)		68.2 (10.9)	
Residence	Urban	243	67.7%	30 (5.4)	.641	112.4 (14.8)	.556	65.7 (15.3)	.895
	Rural	116	32.3%	29.3 (5.1)		111.7 (9.8)		64.2 (15.2)	
Illness may be affected by climate	Yes	153	42.6%	29.9 (5.1)	1.035	110.9 (13)	1.576	63.8 (15.6)	1.578
	No	206	57.4%	29.3 (5.4)		113.2 (13.5)		66.3 (14.9)	
Illness type (*n* = 189)	Heart-related illnesses	33	9.2%	28.6 (4.8)	1.201	111.8 (13.3)	.827	64 (16.3)	1.733
	Respiratory illnesses& allergy	76	21.2%	29.2 (4.4)		112.3 (12.9)		64.4 (16.3)	
	Vector/ water-borne illnesses	30	8.4%	30.9 (4.4)		108 (13.9)		56.9 (13.5)	
	Other	51	13.9%	29.6 (5.4)		113.3 (13.2)		66.1 (15.3)	
Perception Effect of climate change	Water scarcity	31	8.6%	28.7 (4.3)	1.657	112.5 (16.5)	.881	63.3 (17)	1.450
	Biodiversity	15	4.2%	29.5 (5)		111.5 (12.5)		67.3 (16)	
	Desertification	32	8.9%	30.5 (3.8)		113.2 (19.3)		59.0 (14.9)	
	Extreme weather events	143	39.8%	30.2 (6.1)		113.2 (12.6)		66.5 (16)	
	Global warming	125	34.8%	28.6 (4.8)		111.5 (12.1)		65.8 (14.1)	
	Ocean/ River acidification	6	1.7%	30.3 (4.4)		104.3 (6.5)		58.7 (10.4)	
	Sea level rise	7	1.9%	31.4 (4.5)		105.4 (4.6)		68.3 (10.8)	

*EAI* Environmental attitude inventory, *JES* Job engagement scale, *SD* Standard deviation

A notable aspect of the study was the inclusion of participants’ perceptions of illnesses affected by climate change. While a significant portion (57.4%) reported no climate change-related health conditions, the analysis showed no significant differences in climate anxiety, EAI, and JES scores between those who believed their health was affected by climate change and those who did not. Despite the variation in illness types, the results indicated no significant differences in the mean scores of climate anxiety, EAI, and JES across these categories. Finally, participants’ perceptions of the effects of climate change, like water scarcity and desertification, were considered. Although diverse views were presented, these perceptions did not significantly influence the scores of climate anxiety, EAI, and JES.

According to Table 2, the study found that participants with less than 10 years of experience, constituting 53.8% of the sample, exhibited a mean Climate Anxiety score of 29.6 (SD = 5.4), without significant differences compared to those with longer experience. Similarly, the scores for EAI and JES across different experience levels did not show statistically significant variances, suggesting a uniformity in environmental attitudes and job engagement irrespective of the duration of professional experience. Demonstrators, representing 26.2% of the sample, showed mean Climate Anxiety scores of 29.5 (SD = 6.4), which were comparable to those of higher academic ranks such as Assistant Professors and Professors.

**Table 2:** Participants’ professional information and differences regarding Climate anxiety, EAI, and JES

Characteristic	Category	No	%	C Anxiety		EAI		JES	
				Mean (SD)	Sig	Mean (SD)	Sig	Mean (SD)	Sig
Experience	less than 10 years	193	53.8%	29.6 (5.4)	.524	113 (13.9)	.787	65.7 (15)	.714
	10 years to less than 20 years	124	34.5%	29.2 (4.9)		111.2 (12.9)		65.2 (15.3)	
	20 years and more	42	11.7%	30.1 (5.9)		111.3 (11.8)		63.5 (16.9)	
Position	Demonstrator	94	26.2%	29.5 (6.4)	.792	112.8 (12.2)	.800	67.8 (14.2)	1.773
	Assistant lecturer	83	23.1%	29.0 (4.6)		114 (13.2)		66.4 (15.1)	
	Lecturer	94	26.2%	29.3 (5.2)		112 (13.7)		65.2 (14.1)	
	Assistant Professor	46	12.8%	30.5 (4.7)		109.9 (15.7)		62.6 (17)	
	Professor	31	8.6%	29.9 (4.1)		110 (10.5)		60.1 (16.9)	
	Professor Emeritus	11	3.1%	31.2 (4.7)		112.1 (17.2)		61.0 (20)	
Department	Pediatric Nursing	36	10.0%	30.7 (4.9)	.557	114.4 (13.7)	.384	57.7 (18.5)	1.950
	Community Health Nursing	43	12.0%	29.7 (7.4)		112 (12.9)		65.3 (13.7)	
	Medical and Surgical Nursing	64	17.8%	29.7 (5.4)		111.1 (10.1)		67.1 (15.6)	
	Critical Care and Emergency Nursing	49	13.6%	28.5 (4.7)		112.3 (13.6)		67.1 (14.3)	
	Obstetrics and Gynecology Nursing	51	14.2%	29.9 (5.8)		110.8 (11.3)		66.1 (15.9)	
	Nursing Administration	36	10.0%	29.3 (4.6)		113.9 (15.3)		61.3 (15)	
	Gerontological Nursing	25	7.0%	29.2 (4.6)		112.6 (16.4)		67.9 (15)	
	Psychiatric Nursing and Mental Health	38	10.6%	29.5 (4.2)		111.3 (16.3)		68.1 (13.2)	
	Nursing Education	17	4.7%	29.1 (3.2)		114.3 (14.7)		64.1 (12.7)	
University	Cairo	52	14.5%	28.9 (4.7)	4.274 ***	111.6 (11.8)	.877	68.2 (14.2)	3.314 **
	MTI	19	5.3%	29.3 (4)		111.7 (9.5)		69.7 (17.9)	
	Mansoura	61	17.0%	27.7 (5.1)		113.9 (10.8)		70.5 (14.6)	
	Damanhur	39	10.9%	28.8 (6.1)		109.9 (10.8)		66.7 (16.8)	
	Kafr-elsheikh	22	6.1%	31.0 (7.5)		112.3 (13.6)		66.9 (13.4)	
	Alexandria	99	27.6%	29.8 (4.6)		110.8 (16.7)		61.2 (13.9)	
	Port Said	23	6.4%	28.4 (4.4)		112.7 (14.9)		61.6 (17)	
	Beni Suef	44	12.3%	32.8 (5.1)		115.5 (12.3)		61.5 (15)	
University location	Center	71	19.8%	29.2 (4.5)	5.264 ***	111.6 (11.2)	.973	68.6 (15.2)	5.447 ***
	Delta	124	34.5%	28.7 (5.9)		112.3 (11.3)		68.6 (15)	
	West	96	26.7%	29.8 (4.6)		110.9 (16.9)		61.0 (14)	
	East and Suez Canal	23	6.4%	28.4 (4.4)		112.7 (14.9)		61.6 (17)	
	Upper Egypt	45	12.5%	32.6 (5.2)		115.5 (12.2)		61.7 (14.9)	

*EAI* Environmental attitude inventory, *JES* Job engagement scale, *SD* Standard deviation

^**^The correlation is significant at the 0.01 level. ^***^The correlation is significant at the 0.001 level

Departments representing specialties within the nursing faculties also did not exhibit significant disparities in terms of Climate Anxiety, EAI, and JES scores. For example, participants from Pediatric Nursing showed a slightly higher mean score in Climate Anxiety (30.7, SD = 4.9) compared to those in Medical and Surgical Nursing (29.7, SD = 5.4). Participants from the University of Cairo, for example, displayed distinct patterns in Climate Anxiety and JES scores. Such findings suggest that the university environment might contribute to varying levels of climate anxiety and job engagement among nursing staff.

According to Table 3, Climate Anxiety exhibited a mean score of 29.54 with a standard deviation of 5.26. It was found to have modest, yet statistically significant, positive correlations with the EAI total score (*r* = 0.150, *p* < 0.01). Further examination revealed that Climate Anxiety showed negative correlations with all dimensions of JES (Physical, Emotional, and Cognitive) as well as the total score of the JES. Specifically, the correlations with Physical Engagement (*r* = -0.136, *p* < 0.01), Emotional Engagement (*r* = -0.141, *p* < 0.01), Cognitive Engagement (*r* = -0.161, *p* < 0.01), and the total JES score (*r* = -0.156, *p* < 0.01) were statistically significant. In the realm of environmental attitudes, Environment Preservation and Environment Utilization displayed a robust positive correlation (*r* = 0.591, *p* < 0.01), and both were significantly correlated with the EAI total score (Environment Preservation: *r* = 0.829, *p* < 0.01; Environment Utilization: *r* = 0.757, *p* < 0.01). The correlations among the different dimensions of job engagement (Physical, Emotional, and Cognitive) were highly significant and positive, indicating that these aspects of engagement are interrelated and tend to move in tandem.

**Table 3:** Correlations between studied variables *n* = 359

Variables		Mean ± SD	Environment Preservation	Environment Utilization	EAI total	Physical Engagement	Emotional Engagement	Cognitive Engagement	JES total
Climate Anxiety	r	29.54 ± 5.26	.093	.071	.150^b^	-.136^b^	-.141^b^	-.161^b^	-.156^b^
Environment Preservation	r	33.70 ± 4.37	1	.591^b^	.829^b^	.031	-.089	-.067	-.046
Environment Utilization	r	23.03 ± 3.72		1	.757^b^	-.088	-.124^a^	-.108^a^	-.115^a^
EAI total	r	112.2 ± 13.35			1	-.114^a^	-.193^b^	-.178^b^	-.174^b^
Physical Engagement	r	22.46 ± 5.35				1	.798^b^	.763^b^	.914^b^
Emotional Engagement	r	21.44 ± 5.98					1	.852^b^	.952^b^
Cognitive Engagement	r	21.34 ± 5.03						1	.931^b^
Job Engagement Scale total	r	65.24 ± 15.27							1

*EAI* Environmental attitude inventory, *JES* Job engagement scale

^a^The correlation is significant at the 0.05 level

^b^The correlation is significant at the 0.01 level

Table 4 presented a hierarchical multiple regression analysis, which was conducted for variables that showed significant correlation, to examine the relationship between climate anxiety, environmental attitude, and job engagement among Nursing University Colleagues that shown a significant correlation. Demographic characteristics were not included in the analysis because they did not have an impact on the dependent variable. The results indicated that there was no multicollinearity issue in the data, as indicated by the tolerance and variance inflation factor (VIF) values. The analysis was structured into two models to progressively examine the impact of these independent variables on job engagement. The hierarchical approach in the regression analysis allowed for a nuanced understanding of how each variable independently and collectively influenced job engagement, contributing valuable insights into the interplay between personal environmental concerns and professional commitment.

**Table 4:** Multiple linear regression analysis related to job engagement of the studied Nursing University Colleagues (*N* = 359)

Independent variable	Model 1					Model				
	B	SE (B)	β	*t*	p	B	SE (B)	β	*t*	p
Climate anxiety	-0.45	0.15	-0.16	-2.99	0.003	-0.39	0.15	-0.13	-2.54	0.11
Environmental attitude						-0.18	0.06	-0.15	-2.95	0.003
*F* (p)	8.913 (0.003)					8.901 (< 0.001)				
*R* ^2^	0.0224					0.048				
Adjusted *R* ^2^	0.022					0.042				
Constant	78.613					96.427				

*SE* Standard error, *β* standardized regression coefficient

In Model 1, which included only climate anxiety as the independent variable, the regression analysis revealed that climate anxiety negatively influenced job engagement. Specifically, the coefficient for climate anxiety was -0.45, with a standard error of 0.15, yielding a standardized regression coefficient (β) of -0.16. This model indicated that climate anxiety was a significant predictor of job engagement (t = -2.99, *p* = 0.003). The R-squared value for this model was 0.0224, suggesting that climate anxiety explained approximately 2.24% of the variance in job engagement among the study participants.

Model 2 extended the analysis by including environmental attitude as an additional independent variable. In this model, both climate anxiety and environmental attitude were found to have significant negative impacts on job engagement. The coefficient for climate anxiety slightly decreased to -0.39 (SE = 0.15, β = -0.13, t = -2.54, *p* = 0.11), while the coefficient for environmental attitude was -0.18 (SE = 0.06, β = -0.15, t = -2.95, *p* = 0.003). The inclusion of environmental attitude improved the explanatory power of the model, with an R-squared value of 0.048, indicating that these two variables collectively accounted for 4.8% of the variance in job engagement. The results from these regression analyses were instrumental in highlighting the negative impacts of climate anxiety and environmental attitudes on job engagement in our study population. This indicated that higher levels of climate anxiety and stronger environmental attitudes were associated with lower levels of job engagement among Nursing University Colleagues.

## Discussion

Climate anxiety and environmental attitudes wield significant influence as determinants of job engagement. The comprehension and mitigation of climate anxiety, coupled with the cultivation of favorable environmental attitudes among Nursing University Colleagues, stand as pivotal measures contributing to the enhancement of their job engagement and holistic well-being [11, 24].

The present investigation scrutinized the association between anxiety related to climate change, environmental attitude, and academic job engagement among Nursing University Colleagues. The results presented that demographic characteristics had no significant impact on climate anxiety, environmental attitude, or job engagement.

The fact that age, gender, marital status, and residence do not exert influence on climate anxiety, environmental attitudes, or job engagement, suggests that the emotional and attitudinal dimensions linked to climate change and environmental matters remain relatively consistent across a diverse range of demographic groups. This finding indicates a commonality in how individuals from various age brackets, genders, marital statuses, and geographical locations respond to issues related to climate. Such consistency underscores the importance of adopting a comprehensive and inclusive approach when developing interventions and policies, recognizing the universal nature of climate concerns. It emphasizes the necessity for collective efforts that resonate with a broad cross-section of the population, irrespective of individual demographic characteristics.

However, regional differences were observed, with participants from Upper Egypt reporting higher climate anxiety and lower job engagement compared to those from the central and Delta regions of Egypt. There were significant negative correlations between climate anxiety and job engagement dimensions, as well as between environmental attitude and job engagement dimensions. Hierarchical multiple regression analysis revealed that climate anxiety negatively influenced job engagement, and both climate anxiety and environmental attitude together clarified a significant portion difference in job engagement related to Nursing University Colleagues. These findings suggest that addressing climate anxiety and promoting positive environmental attitudes may be important for enhancing job engagement in this population.

The observed regional differences in climate anxiety and job engagement among participants in Egypt reveal the potential influence of various localized factors. The higher levels of climate anxiety reported by individuals from Upper Egypt may stem from distinct environmental challenges specific to that region, such as increased vulnerability to extreme weather events, water scarcity, or economic disparities and resource access inequalities could also contribute to heightened climate anxiety in this area. Furthermore, the lower job engagement reported by participants from Upper Egypt might be linked to regional lower economic conditions, lack of employment opportunities, or job stability. The central and Delta regions may benefit from more favorable environmental conditions or economic prospects, leading to lower climate anxiety and higher job engagement.

In consonance with antecedent scholarly inquiries [11, 25]. Ogunbode study in 2022 [11], of climate change across 32 countries, discovered a significant negative correlation between anxiety related to climate change and psychological well-being, alongside a correlation in positive way between anxiety related to climate change and both co-environmental behavior and environmental activism. The aforementioned findings indicate that climate anxiety could potentially hinder individuals' participation in environmental initiatives and constitute a risk to their overall well-being.

A scholarly inquiry orchestrated by Lukacs and associates in the year 2023, indicated that in 1553 participants, elevated scores in climate change anxiety and increased engagement in climate-related behaviors were both linked to higher levels of psychological distress. An interaction analysis revealed that as climate change anxiety scores rose, the impact of self-reported behavioral engagement on psychological distress diminished. The phenomenon under discussion is commonly known as "the concerned steward effect," which pertains to the inclination of persons who possess a higher level of worry towards climate change to actively take part in actions aimed at mitigating climate impacts [10]. Nevertheless, it was noted that this phenomenon was less pronounced among persons who experienced elevated degrees of anxiety related to climate change.

Regarding the high levels of anxiety related to climate change observed among Egyptian participants, Hajek and König (2023) found a similar higher climate anxiety level [12]. Another Egyptian study by Elsharkawy, Elsheikh, and Refaat in 2023 highlighted the high awareness of climate change among Egyptians and its association with general mitigation measures [26].

Daeninck, Kioupi, and Vercammen (2023) conducted an inquiry into climate anxiety, coping strategies, and future planning among students pursuing environmental degrees. Their findings indicate that environmental degree students exhibit elevated levels of climate anxiety, engage more frequently in coping strategies, and are actively considering career paths that prioritize addressing climate change. Notably, the study revealed a correlation between the use of problem-focused coping strategies and higher rates of climate anxiety. This implies that despite the limited direct exposure to climate change impacts compared to more vulnerable regions, university students in the UK are actively involved in the issue, showcasing a commitment to addressing climate concerns through their academic pursuits and career aspirations [5].

Given that Egypt is susceptible to climate change differences impacts such as temperature elevation, water scarcity, and weather unbalanced incidents, these environmental challenges, coupled with limited resources and infrastructure, may contribute to heightened concerns and anxiety about climate-related issues [26]. Additionally, socio-economic factors like income disparities and access to information and education can also influence levels of climate anxiety [18, 27].

The observed differences in climate anxiety and job engagement between participants from Upper Egypt and those from the central and Delta regions of Egypt may be influenced by a range of factors. Regional disparities in environmental conditions and climate-related challenges could contribute to varying levels of climate anxiety. For example, Upper Egypt experiences higher temperatures, water scarcity, and agricultural challenges compared to other regions, which may heighten climate anxiety among its residents. Socioeconomic factors such as income disparities, access to resources, and educational opportunities could also play a role in shaping climate anxiety levels and job engagement. Limited resources and infrastructure in certain regions may contribute to lower job engagement among participants from Upper Egypt [28, 29].

Regional disparities and environmental challenges have been identified as influential factors affecting psychological well-being and job engagement, as noted by Helbich (2018) who found higher levels of psychological distress among residents in areas with environmental challenges [30]. International Monetary Fund (2023) also highlighted the impact of socioeconomic factors on job engagement in Egypt, emphasizing the significance of income disparities and access to education [31].

The outcomes of our investigation have illuminated a substantive correlation, providing insightful revelations into the nuanced interrelationship between anxiety related to climate change and the total score of environmental attitudes among university staff. This correlation underscores the intricate interplay between psychological well-being and environmental consciousness in the aspect of climate change. Our study expands upon existing research by providing empirical evidence that a more positive environmental attitude is associated with lower levels of climate anxiety, suggesting that individuals who exhibit a greater concern for environmental issues tend to experience reduced anxiety related to climate change.

Innocenti et al., and Zacher & Rudolph [9, 32] support the positive correlation between climate anxiety and environmental attitudes, suggesting that individuals experiencing heightened levels of climate anxiety tend to exhibit more positive attitudes toward the environment. This may be attributed to the perception of a more threatened environment leading to increased environmental concern and responsibility [33]. Additionally, individuals with high climate anxiety may adopt positive environmental attitudes as a cognitive coping strategy to manage their anxiety and regain a sense of control [4]. Increased awareness of climate change influences and the urgency of addressing environmental issues have also been linked with higher levels of both climate anxiety and environmental concern [24].

The present findings align coherently with the research conducted by Clayton et al. (2023), who found a similar relationship between environmental attitudes and emotional reactions to climate differences in a varied sample of urban residents [34]. However, our study focused specifically on Nursing University Colleagues, a unique population that has received limited attention with anxiety related to climate change. This perspective is particularly consistent with the role of education and knowledge dissemination within the academic community. Our findings suggest that fostering a positive environmental attitude could serve as a potential coping mechanism to mitigate climate anxiety, and this insight can be leveraged to design interventions aimed at enhancing both emotional well-being and environmental consciousness.

Inversely, the negative correlation between climate anxiety and different proportions of job engagement, including physical, affective, and thoughtful engagement, as well as overall academic job engagement, suggests that higher levels of climate anxiety are accompanied by decreased engagement in various aspects of work. Climate anxiety can lead to increased psychological distress, divert attention from work-related tasks, and evoke feelings of helplessness or despair, all of which can undermine individuals' ability to fully engage in their work [8, 10, 35].

The hierarchical multiple regression prediction indicates that climate anxiety and environmental attitude significantly predict job engagement among Nursing University Colleagues. Climate anxiety may hinder individuals' job engagement due to increased distress and worry associated with climate-related issues, while positive environmental attitudes can shape individuals' values and beliefs, influencing their motivation and commitment to their work [11, 24, 36].

Furthermore, the effect of climate difference-related physical illnesses on emotional well-being and job engagement should not be underestimated. The increasing prevalence of physical illnesses caused or exacerbated by anxiety related to climate change has adverse effects on individuals' work productivity, absenteeism, and overall job satisfaction [25, 37–39].

Comparatively, these findings align with Donley [40], who reported a negative association between ecological distress and job satisfaction in a corporate setting. However, our study advances the literature by not only focusing on job satisfaction but also comprehensively investigating the nuanced dimensions of job engagement in an academic environment. Our research highlights the far-reaching implications of climate anxiety and environmental attitude on not only psychological well-being but also on the degree to which individuals are connected and invested in their work roles.

The significant negative correlations observed in our study provide valuable insights into the potential impact of climate anxiety and environmental attitudes on job engagement among Nursing University Colleagues. The negative relationship suggests that higher levels of climate anxiety accompany reduced engagement in the cognitive, emotional, and physical aspects of one's job. Similarly, a more positive environmental attitude is associated with diminished job engagement across these dimensions. This raises important questions about the mechanisms through which climate anxiety and environmental attitudes influence job engagement. Attitude influences job engagement.

In summary, the elucidation of regional disparities, environmental challenges, climate anxiety, and the intricate interplay among environmental attitudes, job engagement, and physical illnesses collectively emerge as pivotal constituents in comprehending the nuanced and multifaceted dynamics inherent in the interaction between climate change, mental well-being, and work-related outcomes. This comprehensive analysis encapsulates the intricate relationships among these elements, shedding light on their interconnectedness and providing a holistic perspective for scholarly and practical considerations within the ambit of climate change impacts on mental health and professional endeavors.

### Implication and strengths

This study, in its totality, holds the potential to enrich the extant scholarly corpus by advancing a nuanced understanding of the interplay between anxiety associated with climate change, environmental attitudes, and job engagement within the context of Nursing University Colleagues, employing a cross-cultural lens in the Egyptian milieu. The discerned findings not only furnish valuable insights for practitioners, policymakers, and administrators involved in climate change and sustainability education within Egyptian universities but also proffer broader implications for the formulation of educational strategies.

The implications of this research extend to policymakers as they deliberate upon the formulation of university and national-level policies pertaining to climate change education. The findings offer substantive grounds for the development of legal frameworks at the local level, thereby fortifying support for climate change education interventions across various educational tiers, with a particular emphasis on higher education institutions.

Furthermore, the administrators of universities can leverage these insights to inform their decision-making processes. Specifically, the application of psycho-educational interventions emerges as a viable strategy for mitigating climate anxiety, fostering adaptive capacities, and nurturing mental well-being, consequently enhancing job engagement among nursing university colleagues. This strategic approach, grounded in empirical evidence, underscores the potential for universities to play a transformative role in enhancing the psychological resilience and professional commitment of their academic constituents.

Moreover, these findings bear relevance for researchers and scientific practitioners, serving as a springboard for future investigations in the expansive domain of climate change. The gaps identified and insights gleaned from this study pave the way for a more comprehensive exploration of climate-related psychological phenomena, thereby facilitating the ongoing scholarly discourse and advancements in the field.

### Limitation

The study focused on nursing university colleagues, which limits the generalizability of the findings to other healthcare professionals or the general population. Further research with a more diverse sample is needed to validate the findings in different contexts. The research relies on self-reported data using convenience sampling, which may be subject to social desirability bias or inaccuracies. The study's cross-sectional nature, assuming it is a one-time assessment, restricts the ability to find causatives or examine changes over time. Longitudinal or repeated measures would offer a systematic comprehension. Future studies could incorporate additional objective measures or observational data to strengthen the validity of the findings.

## Conclusion

In summation, this study compellingly illustrated a robust correlation between climate anxiety, environmental attitudes, and job engagement within the cohort of nursing university colleagues. Elevated levels of climate anxiety exhibited a discernible association with diminished environmental attitudes, concurrently leading to a decrease in job engagement. Concomitantly, a heightened disposition toward the environment was found to be inversely related to the overall engagement in participants' job roles.

Furthermore, the spatial distribution of universities and participants' affiliations illuminated noteworthy regional disparities in both climate anxiety and job engagement. These geographical variations underscore the nuanced interplay of contextual factors that contribute to divergent levels of climate-related distress and professional commitment across distinct regions. Such discernments offer valuable insights for tailored interventions and region-specific strategies aimed at fostering psychological well-being and enhancing job engagement among nursing university colleagues in diverse geographical contexts.

### Supplementary Information

**Additional file 1.**

**Additional file 2.**

## Data Availability

Upon request for scientific purposes, the researcher of correspondence will provide researchable information of the research.

## References

[1] Leiserowitz A, Carman J, Buttermore N, Wang X, Rosenthal S, Marlon JR, Mulcahy K. International public opinion on climate change. Yale program on climate change communication. 2021. Retrieved from: https://climatecommunication.yale.edu/publications/international-public-opinion-on-climate-change/.

[2] Clayton S, Manning CM, Krygsman K, Speiser M. Mental health and our changing climate: impacts, implications, and guidance. American Psychological Association and ecoAmerica: Washington, DC. 2017. ‏ https://www.apa.org/news/press/releases/2017/03/mental-health-climate.pdf.

[3] United Nations Development Programme. Climate promise: a youth-led guide to climate action. 2020. Retrieved from https://www.undp.org/content/undp/en/home/librarypage/climate-and-disaster-resilience-/climate-promise--a-youth-led-guide-to-climate-action.html.

[4] Rawat A., Kumar D., Khati BS. A review on climate change impacts, models, and its consequences on different sectors: a systematic approach. J Water Climate Change. 2023:jwc2023536. 10.2166/wcc.2023.536.

[5] Daeninck C, Kioupi V, Vercammen A (2023). Climate anxiety, coping strategies and planning for the future in environmental degree students in the UK. Front Psychol.

[6] Clayton S, Karazsia BT (2020). Development and validation of a measure of climate change anxiety. J Environ Psychol.

[7] Walinski A, Sander J, Gerlinger G, Clemens V, Meyer-Lindenberg A, Heinz A (2023). The Effects of Climate Change on Mental Health. Dtsch Arztebl Int.

[8] Dodds J (2021). The psychology of climate anxiety. BJPsych Bulletin.

[9] Zacher H, Rudolph CW (2023). Environmental knowledge is inversely associated with climate change anxiety. Clim Change.

[10] Lukacs JN, Bratu A, Adams S, Logie C, Tok N, McCunn LJ, Lem M, Henley A, Closson K, Martin G, Gislason MK, Takaro T, Card KG (2023). The concerned steward effect: Exploring the relationship between climate anxiety, psychological distress, and self-reported climate related behavioural engagement. J Environ Psychol.

[11] Ogunbode CA, Doran R, Hanss D, Ojala M, Salmela-Aro K, van den Broek KL, Karasu M (2022). Climate anxiety, wellbeing, and pro-environmental action: Correlates of negative emotional responses to climate change in 32 countries. J Environ Psychol.

[12] Hajek A, König HH (2023). Climate Anxiety and Mental Health in Germany. Climate.

[13] Zhou F, Zhang N, Mou J (2022). Universities as incubators of innovation: The role of a university playfulness climate in teachers' sustainable teaching innovation. The International Journal of Management Education.

[14] Nassani AA, Alhammad B, Alqahtani RF (2021). The relationship between job characteristics and job satisfaction: The mediating role of work engagement in the private sector organizations in Riyadh. J Adv Soc Sci Humanit.

[15] Wang Y, Derakhshan A, Azari Noughabi M. The interplay of EFL teachers’ immunity, work engagement, and psychological well-being: evidence from four Asian countries. J Multilingual Multicultural Dev. 2022;1–17. 10.1080/01434632.2022.2092625.

[16] Ssekamatte D (2023). The role of the university and institutional support for climate change education interventions at two African Universities. High Educ.

[17] Boluda-Verdú I, Senent-Valero M, Casas-Escolano M, Matijasevich A, Pastor-Valero M (2022). Fear for the future: Eco-anxiety and health implications, a systematic review. J Environ Psychol.

[18] World Bank Group. Climate Risk Country Profile. The World Bank Group, 36. 2021. Retrieved from: https://climateknowledgeportal.worldbank.org/sites/default/files/2021-04/15723-WB_Egypt%20Country%20Profile-WEB-2_0.pdf.

[19] Bujang MA (2021). A Step-by-Step Process on Sample Size Determination for Medical Research. MJMS.

[20] Milfont TL, Duckitt J (2010). The environmental attitudes inventory: A valid and reliable measure to assess the structure of environmental attitudes. J Environ Psychol.

[21] Ajdukovic I, Gilibert D, Fointiat V (2019). Structural confirmation of the 24-item Environmental Attitude Inventory/Confirmación estructural del Inventario de Actitudes Ambientales de 24 ítems. Psychology.

[22] Rich BL, LePine JA, Crawford ER (2010). Job engagement: Antecedents and effects on job performance. Acad Manag J.

[23] Houle SA, Rich BL, Comeau CA, Morin AJS, Morin AJS (2022). The Job Engagement Scale: Development and Validation of a Short Form in English and French. J Bus Psychol.

[24] Whitmarsh L, Player L, Jiongco A, James M, Williams M, Marks E, Kennedy-Williams P (2022). Climate anxiety: What predicts it and how is it related to climate action?. J Environ Psychol.

[25] Cianconi P, Betrò S, Janiri L (2020). The impact of climate change on mental health: a systematic descriptive review. Front Psych.

[26] Elsharkawy SA, Elsheikh AA, Refaat LA. Knowledge, perception, and practices regarding climate change among students of Al-Azhar University for Girls in Cairo. Egypt JPublic Health. 2023;1–10. 10.1007/s10389-023-01901-9.

[27] Abdallah ZA, Wagdy Farag AA (2022). Impact of Awareness Program Regarding Health Consequences of Climate Change on Knowledge, Perception and Daily Life practices among Nursing Students. Egypt J Nursing Health Sci.

[28] AbdEllah RG (2020). Water resources in Egypt and their challenges, Lake Nasser case study. The Egyptian Journal of Aquatic Research.

[29] Nikiel CA, Eltahir EA (2021). Past and future trends of Egypt’s water consumption and its sources. Nat Commun.

[30] Helbich M (2018). Mental health and environmental exposures: An editorial. Int J Environ Res Public Health.

[31] International Monetary Fund. ARAB REPUBLIC OF EGYPT Request for Extended Arrangement Under the Extended Fund Facility—Press Release; And Staff Report. IMF Country Report No. 23/2, Washington, D.C. 2023. Retrieved from: https://www.imf.org/en/Publications/CR/Issues/2023/01/06/Arab-Republic-of-Egypt-Request-for-Extended-Arrangement-Under-the-Extended-Fund-Facility-527849.

[32] Innocenti M, Santarelli G, Lombardi GS, Ciabini L, Zjalic D, Di Russo M, Cadeddu C (2023). How can climate change anxiety induce both pro-environmental behaviours and eco-paralysis? The mediating role of general self-efficacy. Int J Environ Res Public Health.

[33] Tam K-P, Chan H-W, Clayton S (2023). Climate change anxiety in China, India, Japan, and the United States. J Environ Psychol.

[34] Clayton SD, Pihkala P, Wray B, Marks E (2023). Psychological and Emotional Responses to Climate Change among Young People Worldwide: Differences Associated with Gender, Age, and Country. Sustainability.

[35] Fortes AM, Tian L, Huebner ES (2020). Occupational stress and employees' complete mental health: A cross-cultural empirical study. Int J Environ Res Public Health.

[36] Katz IM, Rauvola RS, Rudolph CW, Zacher H (2022). Employee green behavior: A meta-analysis. Corp Soc Responsib Environ Manag.

[37] World Health Organization. Climate change and health. 2023. Retrieved from: https://www.who.int/news-room/fact-sheets/detail/climate-change-and-health.

[38] The Commonwealth Fund. Improving Health Care Quality: The Impact of Climate Change on Our Health and Health Systems. 2022. Retrieved from: https://www.commonwealthfund.org/publications/explainer/2022/may/impact-climate-change-our-health-and-health-systems.

[39] Rocque RJ, Beaudoin C, Ndjaboue R, Cameron L, Poirier-Bergeron L, Poulin-Rheault R-A, Fallon C, Tricco AC, Witteman HO (2023). Health effects of climate change: An overview of systematic reviews. Public Health.

[40] Donley J (2021). The Impact of Work Environment on Job Satisfaction: Pre-COVID Research to Inform the Future. Nurse Lead.

