# Supplementary material for: Climate anxiety, environmental attitude, and job engagement among nursing university colleagues: a multicenter descriptive study
Source: BMC Nurs. 2024 Feb 20;23:133. doi: 10.1186/s12912-024-01788-1 (PMC10880327; doi:10.1186/s12912-024-01788-1)
Supplement: Supplementary file 2 — Additional file 2. [file 12912_2024_1788_MOESM2_ESM.docx]

**Social and Health-related Climate Change questionnaire**

**Part 1: Socio-demographic**

- **Age :**

20 < 30 ( ) 30 < 40 ( ) 40 < 50 ( )

50 < 60 ( ) 60 < 70 ( ) 70 - ( )

- **Sex:**

Male ( ) 2. Female ( )

- **Residence:**

Rural ( ) 2. Urban ( )

- **Marital status.**

Single ( ) Married ( ) Widow ( ) Divorced ( )

**Part 2: Academic profile/ health related problem**

- **Position:**

Demonstrator ( ) Assistant lecturer ( ) lecturer ( )

Assistant professor ( ) professor ( ) Professor Emeritus ( )

- **Department:**

Medical and Surgical Nursing ( ) Critical care and Emergency ( )

Obstetrics and Gynecology Nursing ( ) Pediatric Nursing ( )

Nursing Administration ( ) Nursing Education ( )

Gerontological Nursing ( ) Community Health Nursing ( )

Psychiatric Nursing and Mental Health ( )

- **Name of University:**

Alexandria ( ) Damnhour ( ) Kafrelshekh ( ) MTI ( )

Mansoura ( ) Port said ( ) Beni Suef ( ) Cairo ( )

- **Years of experience:**

1 < 5 ( ) 5 < 10 ( ) 10 < 15 ( )

15 < 20 ( ) 20 < 25 ( ) 30 – ( )

- **Do you have physical illnesses affected by climate? Yes ( ) No( )**

If yes, which type:

Respiratory illnesses& allergy ( ) Heat-related illnesses ( )

Vector/ water-borne illnesses ( ) others ( )…………………..

- **From your point of view, what is the most climate change you know?**

1. Extreme weather events
2. Water scarcity
3. Desertification
4. Global warming.
5. Ocean/ River acidification.
6. Sea level rise.
7. Biodiversity
